# VISTA: A Tool for Fast Taxonomic Assignment of Viral Genome Sequences

**DOI:** 10.1093/gpbjnl/qzae082

**Published:** 2024-11-14

**Authors:** Tao Zhang, Yiyun Liu, Xutong Guo, Xinran Zhang, Xinchang Zheng, Mochen Zhang, Yiming Bao

**Affiliations:** National Genomics Data Center, China National Center for Bioinformation, Beijing 100101, China; Beijing Institute of Genomics, Chinese Academy of Sciences, Beijing 100101, China; University of Chinese Academy of Sciences, Beijing 100049, China; National Genomics Data Center, China National Center for Bioinformation, Beijing 100101, China; Beijing Institute of Genomics, Chinese Academy of Sciences, Beijing 100101, China; Sino-Danish College, University of Chinese Academy of Sciences, Beijing 100049, China; National Genomics Data Center, China National Center for Bioinformation, Beijing 100101, China; Beijing Institute of Genomics, Chinese Academy of Sciences, Beijing 100101, China; University of Chinese Academy of Sciences, Beijing 100049, China; National Genomics Data Center, China National Center for Bioinformation, Beijing 100101, China; Beijing Institute of Genomics, Chinese Academy of Sciences, Beijing 100101, China; University of Chinese Academy of Sciences, Beijing 100049, China; National Genomics Data Center, China National Center for Bioinformation, Beijing 100101, China; Beijing Institute of Genomics, Chinese Academy of Sciences, Beijing 100101, China; National Genomics Data Center, China National Center for Bioinformation, Beijing 100101, China; Beijing Institute of Genomics, Chinese Academy of Sciences, Beijing 100101, China; University of Chinese Academy of Sciences, Beijing 100049, China; National Genomics Data Center, China National Center for Bioinformation, Beijing 100101, China; Beijing Institute of Genomics, Chinese Academy of Sciences, Beijing 100101, China; University of Chinese Academy of Sciences, Beijing 100049, China

**Keywords:** Virus taxonomic assignment, Pairwise sequence comparison, Unclassified virus, Viral genome, Demarcation

## Abstract

The rapid expansion of the number of viral genome sequences in public databases necessitates a scalable, universal, and automated preliminary taxonomic framework for comprehensive virus studies. Here, we introduce Virus Sequence-based Taxonomy Assignment (VISTA), a computational tool that employs a novel pairwise sequence comparison system and an automatic demarcation threshold identification framework for virus taxonomy. Leveraging physio-chemical property sequences, *k*-mer profiles, and machine learning techniques, VISTA constructs a robust distance-based framework for taxonomic assignment. Functionally similar to Pairwise Sequence Comparison (PASC), a widely used virus assignment tool based on pairwise sequence comparison, VISTA demonstrates superior performance by providing significantly improved separation for taxonomic groups, more objective taxonomic demarcation thresholds, greatly enhanced speed, and a wider application scope. We successfully applied VISTA to 38 virus families, as well as to the class *Caudoviricetes*. This demonstrates VISTA’s scalability, robustness, and ability to automatically and accurately assign taxonomy to both prokaryotic and eukaryotic viruses. Furthermore, the application of VISTA to 679 unclassified prokaryotic virus genomes recovered from metagenomic data identified 46 novel virus families. VISTA is available as both a command line tool and a user-friendly web portal at https://ngdc.cncb.ac.cn/vista.

## Graphical abstract

**Figure:**
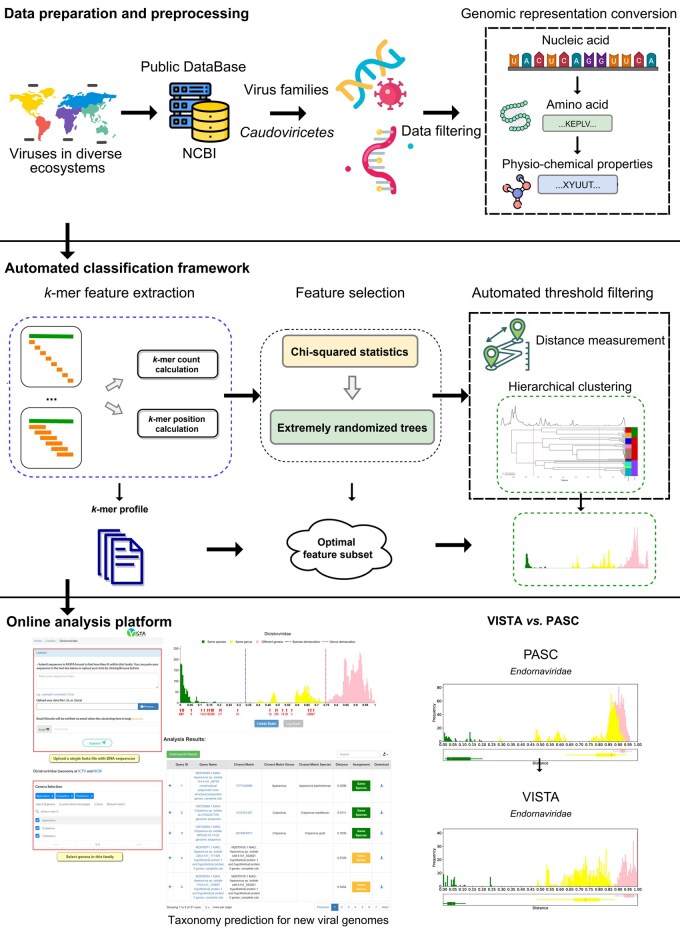

## Introduction

Viruses, among the most prevalent and diverse microscopic entities on Earth, exert a significant influence on the biosphere [1–3]. Virus taxonomy is a scientific field that focuses on characterizing the properties and attributes of viral entities to establish a systematic classification framework that accurately reflects their evolutionary relationships. Accurate taxonomic assignment of viruses is crucial for understanding virus diversity, evolution, and potential pathogenicity. Traditionally, virus taxonomic assignment relied on viral biological properties such as virion morphology, structure, host tropisms, pathogenicity, and replication mechanisms, which required sampling and cultivation of viruses for experimental validation [4,5]. However, the majority of viruses are not readily cultivable under normal laboratory conditions [6], resulting in a gross underestimation of the global virus diversity and a non-systematic taxonomy landscape [7,8]. In recent years, shotgun metagenomic sequencing has revealed extensive diversity of uncultivated viruses in various environmental samples and host organisms [9,10], contributing to a rapid expansion in the number of available viral genomes in public databases. Considering the limited availability of phenotypic attributes for taxonomic assignment of viral genomes derived from metagenomics, the International Committee on Taxonomy of Viruses (ICTV) now recognizes genomes that have undergone strict sequence quality control as actual viruses and allows their inclusion in the official classification system [5,11].

To satisfy the growing need for virus taxonomic assignment based solely on genome sequences, an increasing number of computational tools have been developed. Phylogenetic analysis, a popular method for virus taxonomic assignment, constructs highly resolved phylogenetic trees to infer evolutionary relationships among virus sequences by aligning conserved proteins (*e.g.*, capsid protein and portal protein) [12,13]. However, phylogenetic inference is notoriously time-consuming, especially for large datasets [14], and requires expertise to demarcate monophyletic groups as taxa. Another commonly used approach is to apply a clustering algorithm (*e.g.*, Markov clustering) to viruses based on shared genes between member viruses within a taxon group. For eukaryotic viruses, GRAViTy [15] assigns a virus to a reference taxonomic group at the family rank using gene signatures and genome organizational features. For prokaryotic viruses (archaeal and bacterial viruses), vConTACT (v.2.0) [16] utilizes gene-sharing networks with confidence scores, enabling the taxonomic assignment of viruses at the genus, subfamily, and family ranks. Although these tools demonstrate high sensitivity and specificity in taxonomic assignment, they are limited to certain viruses and do not support the estimation of virus taxa at lower ranks (*e.g.*, species).

Pairwise sequence comparison, the popular sequence-based assignment approach used by the ICTV, is steadily gaining popularity for assisting decision-making in virus taxonomy [17–19]. Several tools are available for obtaining pairwise sequence divergence between viruses, including Pairwise Sequence Comparison (PASC) [20–22], DEmARC [23], SDT [24], VICTOR [25], and VIRIDIC [26]. Among them, PASC, a web-based tool developed by the National Centre for Biotechnology Information (NCBI), was the first to be used for virus taxonomic assignment and has a broad utility in virology studies. PASC performs two rounds of BLAST [27] on each pair of genome sequences to identify conserved regions, from which the pairwise identities are calculated and their distributions are plotted to help establish demarcation criteria within a virus family. PASC has been successfully applied to many virus families, and its analysis results for several virus families have been adopted by the ICTV Study Groups [28–33]. However, PASC fails to deal with virus families with highly diverse or distantly related sequences, including those with low overall sequence identities or huge differences in genome length and organization. Furthermore, the determination of taxonomic demarcation using PASC remains subjective and heavily relies on manual analysis and curation, leading to biases and inefficiencies that impede the establishment of taxonomic assignment standards for many viruses. Lastly, the phage taxonomy was updated by ICTV in August 2022, in which the order *Caudovirales* and the phage families *Myoviridae*, *Podoviridae*, and *Siphoviridae* in the previous ICTV system were abolished. All former members of these taxa were automatically reassigned to the class *Caudoviricetes*, resulting in numerous genera becoming free floating. Due to the lack of clear family demarcation criteria, dozens of genera were assigned to temporary subfamilies. However, this reorganization still left a large number of viral sequences unclassified within the *Caudoviricetes*, presenting a challenge for their taxonomic assignment [34,35]. Currently, PASC has not incorporated these changes to accommodate the latest ICTV classification framework, and the taxonomic assignments of sequences in its database are outdated, which could potentially mislead users.

To address these problems, we present Virus Sequence-based Taxonomy Assignment (VISTA), which shares functional similarities with PASC but features a novel pairwise sequence comparison system and a computational framework for automatic identification of demarcation thresholds. Together, these features enable higher speed, higher automation, and greatly improved taxonomic assignment compared to PASC for both prokaryotic and eukaryotic viruses. We have successfully applied our approach to 38 virus families, as well as to the class *Caudoviricetes*. The results demonstrate that VISTA is robust and scalable.

## Method

### Datasets

Complete genome sequences of viruses belonging to a specific family were downloaded from the NCBI Viral Genomes Resource [36] in FASTA format, which comprised both reference sequences and genomes of other members of the same species (January 2022). Following the retrieval of viral sequences, filtering steps were applied to further exclude incomplete genome sequences and reduce redundancy in the dataset. The description line (defline) of each sequence data record was examined. Sequences containing any of the following keywords, namely “partial”, “ORF”, “CDS”, “gene”, “protein”, and “region”, were subsequently excluded from the dataset. Additionally, sequences with PASC identities higher than 99.5% were considered to be redundant and represented by only one sequence in the dataset. After the filtrations, the final dataset comprised 21,263 complete viral genome sequences, covering 6 Baltimore classes, 1 class (*Caudoviricetes*), and 38 additional virus families (Table S1).

### Physio-chemical property sequences

We first considered the nature of each nucleotide sequence, whether it was linear or circular. For each circular genome sequence, we treated it as a linear sequence by concatenating it with itself, thereby creating a duplicated sequence. This step ensured that no potential open reading frames (ORFs) were missed due to artificial breaks. Linear genome sequences were left unaltered. Next, a standard six-frame translation method was performed using the sixpack package from the EMBOSS suite [37], with methionine (M) as the start codon for translation initiation. The translated sequences were split at every stop codon, and any sequences shorter than a predefined threshold of 100 amino acids were removed. This conservative length cutoff helped reduce the false-positive discovery rates for viral proteins [15]. After translation, we removed redundant ORFs resulting from the concatenation step for circular genomes. Subsequently, we cataloged the 20 amino acids into 7 classes based on their dipoles and volumes of the side chains [38,39]. These 7 physio-chemical properties were represented by 7 different letters, which were used to constitute physio-chemical property sequences. Finally, the *k*-mers were extracted using these 7 letters as the alphabet.

### *k*-mer profiles

*k*-mer counts were computed using the alfpy package (v1.0.6) [40]. To take into account the significance of genomic organization in the taxonomy of viruses, *k*-mer position was recorded as well. The combination of *k*-mer counts and *k*-mer position distribution was utilized to construct *k*-mer profiles. This integrated approach we proposed aims to offer a more comprehensive characterization of the viral genomes.

The number of times for each particular word (subsequences of length k) occurs in each ORF was computed separately and then summed, yielding the total *k*-mer counts of a sequence. Consider a sequence $S=\left\{s_{i}\right\}$, i = 1 to *N*, which represents the sequence that has *N* ORFs. Given a word size of k, *k*-mer counts of the physio-chemical property sequence S can be represented by a $7^{k}$-dimensional vector as follows:


(1) $$<n_{S,W}>\;=\;<\sum_{i=1}^{N}n_{s_{i}{,w}_{1}},\sum_{i=1}^{N}n_{s_{i}{,w}_{2}},\ldots ,\sum_{i=1}^{N}n_{s_{i},w_{7^{k}}}>$$

where W is the set of all theoretically possible *k*-mers generated by the sequence S. Next, to locate each *k*-mer within a genome sequence, ORFs were concatenated to form a single sequence. For a sequence S, let $w_{k}$ be the k-th element of the *k*-mer set W and $P_{{S,w}_{k}}$ be the position vector of $w_{k}$ of sequence S. Suppose n is the count of $w_{k}$ in the sequence S. We defined the position distribution of $w_{k}$ as follows:


(2) $$D_{S,w_{k}}=\{\begin{array}{cc} \frac{\sum_{i=2}^{n}\left(P_{S,w_{k,i}}-P_{S,w_{k,i-1}}\right)+P_{S,w_{k,1}}}{\sum_{i=1}^{n}P_{S,w_{k,i}}}, & n>0\\ \;\;\;\;\;\;\;\;\;\;\;\;\;\;\;\;\;\;\;\;\;\;\;\;\;\;\;\;\;\;\;\;\;\;\;\;\;\;\;\;\;\;\;\;\;\;\;\;\;\;\;\;\;\;\;\;\;\;\;\;\;\;\;\;\;\;\;\;\;\;\;\;\;\;\;0, & n=0 \end{array}$$

where $P_{{S,w}_{k,i}}$ is the i-th element of position vector $P_{S,w_{k}}$. The position distribution considered the average relative positioning and spacing between each *k*-mer instead of absolute positions. Likewise, the *k*-mer position distribution of sequence S can be represented by a $7^{k}$-dimensional vector as follows:


(3) $$<D_{S,W}>=<D_{{S,w}_{1}},D_{{S,w}_{2}},\ldots ,D_{S,w_{7^{k}}}>$$

The *k*-mer count vector and the *k*-mer position distribution vector were concatenated to create the final feature vector space. To identify the optimal *k*-mer length suitable for pairwise genome comparison, a range of *k*-mer lengths ranging from 1 to 6 was evaluated across various virus families, including different types and sizes of genomes (Figure S1A). The performance appeared to improve with increasing *k*-mer length and began to stabilize at k=5. Moreover, combinations of different *k*-mer lengths ($k\in \left[3,5\right]$ or $k\in \left[3,6\right]$) were explored to capture a more comprehensive representation of genome sequences.

### Relative *k*-mer matrix

To obtain a subset of features that best relates to taxonomic relationships (*i.e.*, same species, different species but within the same genus, or different genera) within a virus family, *k*-mer profiles need to be converted into relative *k*-mer matrices before performing feature selection. Consider one virus family, which contains N genome sequences $\left\{S_{1},S_{2},\ldots ,S_{N}\right\}$. To obtain the relative *k*-mer matrix, we carried out the pairwise computation of all sequences in the family by calculating the absolute difference between corresponding elements of each vector pair. Our algorithm is defined as follows:


(4) $$R_{i,j}=|S_{i}-S_{j}|=<|n_{S_{i},W}-n_{S_{j},W}|,|D_{S_{i},W}-D_{S_{j},W}|>,\;1\leq i<j\leq N$$

After the transformation, a large increase in the number of rows is expected, *i.e.*,


(5) $$N\to C_{N}^{2}=\frac{N\left(N-1\right)}{2}$$

Therefore, before the transformation, a representative sampling strategy and an unsupervised feature filtering technique were implemented, aiming to reduce memory requirements and mitigate overfitting. The representative sampling approach involved two steps. (1) Given that the viral RefSeq provides high-quality manually curated reference genome records [36], all RefSeq sequences were first extracted from the dataset. (2) Furthermore, five additional sequences were randomly selected for each virus species to avoid sampling bias. If a virus species group contained fewer than five sequences, all available sequences in that group were selected. This subsampling process allowed us to establish a representative training dataset that captures the diversity and complexity of virus species, which is helpful to create a more robust and generalizable model. Following the representative sampling step, the features achieving a variance close to zero across all samples were filtered using the nearZeroVar function from the R caret package with default settings.

### Feature selection

Feature selection methods were used to identify a subset of features that are best predictive of taxonomic relationships of virus sequences within the current ICTV taxonomy. This process involved removing features that were redundant or irrelevant to the investigated virus family.

To address the issue of features having different magnitudes, a min-max normalization method was applied. This normalization technique involved scaling each feature vector to a range of 0 to 1. By doing so, the features were adjusted to a common scale, enabling fair comparisons and preventing any particular feature from dominating the analysis due to its larger magnitude. Consider a feature vector X, the min-max normalization was calculated by the formula:


(6) $$X^{\prime }=\frac{X-\min(X)}{\max\left(X\right)-\min(X)}$$

Afterward, the chi-squared statistic and the extremely randomized trees (ERT) were combined and implemented in Python using the scikit-learn package (v1.0.2). This combination aimed to leverage the strengths of different feature selection algorithms, resulting in improved performance and obtaining more accurate feature sets within a reasonable time frame [41–44]. We first selected the highest scoring percentage of features as the candidate features according to chi-squared values. This univariate feature selection method is computationally efficient but ignoring feature dependencies. Then the more accurate tree-based method, ERT, was used along with the SelectFromModel module in the scikit-learn package to refine the candidate feature set further. All hyperparameters (*e.g.*, percentile, n_estimators, max_features, and threshold) were optimized by 5-fold cross-validation using the GridSearchCV function.

### Distance estimation

The optimal subset of features extracted from *k*-mer profiles was utilized as input to calculate pairwise measures of distance among all genome sequences within the virus family. In total, 19 different distance measures were applied for distance estimation. Among them, 10 measures (Angled-based composition, Angled-based evolutionary distance, Bray-Curtis, Canberra, Euclidean, Jensen-Shannon Divergence, Normalized Google, Mean absolute difference, Manhattan, and Minkowski) were implemented using the alfpy package (v1.0.6) [40], and the remaining 9 measures (Correlation, Dice, Jaccard-Needham, Kulsinski, Rogers-Tanimoto, Standardized Euclidean, Sokal-Michener, Sokal-Sneath, and Squared Euclidean) were implemented using the SciPy package. To ensure consistency and comparability, the final distances obtained from these measures were normalized to the interval [0, 1] using the min-max normalization method.

### Automatic optimal threshold identification

To identify thresholds in a distance distribution and determine the optimal distance measure for each virus family, a Gaussian kernel density estimation was applied to the pairwise distance values. Kernel density estimation is a nonparametric method that allows unbiased estimation of distribution characteristics using sample data, without requiring any prior assumptions about the underlying distribution. By way of example, in this process, the input distance values ${\{x}_{1},x_{2},\ldots ,x_{n}\}$ can be estimated by the Gaussian kernel density estimation as:


(7) $$\hat{p}(x)=\frac{1}{nh\sqrt{2\pi }}\sum_{i=1}^{n}e^{-({x-x_{i})}^{2}/2h^{2}}\;$$

where $\hat{p}\left(x\right)\;$denotes the estimated probability density function of the random variable x, and h is a smoothing parameter called the bandwidth, which controls the trade-off between bias and variance. To optimize the bandwidth, 5-fold cross-validation was performed using the GridSearchCV function. The bandwidth with the maximum cross-validation score [log-likelihood, $\sum \log\hat{p}(x_{i})$] was selected for the analysis.

Subsequently, all local minima (valleys) in the density distribution were detected, since among them there exist thresholds that separate ranks of classification (peaks). We suppose $d_{i}$ as the density at a distance point i and $d_{M}\;$as its adjacent local neighborhood, where $M=\left\{i-m,\ldots ,i-2,i-1,i+1,i+2,\ldots ,i+m\right\}$; the parameter m is the width of the neighborhood. Let $d_{M_{\min}}\;$be the minimum in $d_{M}$, then the evaluation function for valleys can be expressed as:


(8) valley(i)=1, di≤dMmin0, di>dMmin

To reduce the sensitivity of our model to noise contained in the data, the parameter m was set to 4.

Furthermore, hierarchical clustering was conducted on all intergenomic distance values using average linkage, implemented with the fastcluster Python library [45]. The hierarchical clustering process resulted in the formation of different clusters by applying a tree cut to the dendrogram at each distance threshold. The Fowlkes-Mallows index (FMI) [46] was employed to evaluate the quality of inferred clusters compared to the actual genus/species rank clusters. As a comprehensive clustering metric, the FMI can be obtained by computing the geometric mean of the precision and recall values as:


(9) $$\mathrm{FMI}=\sqrt{P\mathrm{recision}\times \mathrm{Recall}}=\sqrt{\frac{TP}{TP+FP}\times \frac{TP}{TP+FN}}$$

where TP is the number of pairs of virus sequences that are present in the same cluster in both inferred clusters and actual taxonomic groups; FP is the number of pairs that are present in the same cluster in the inferred clusters but not in the actual taxonomic groups; and FN are those pairs that appear together in the actual taxonomic groups, but are separated in the inferred clusters. The FMI scores range from 0 to 1, where a score of 1 indicates a perfect correspondence between each inferred cluster and the taxonomic group. The threshold with the maximum FMI score was selected as the optimal taxonomic demarcation threshold and its corresponding distance measure was chosen for the virus family.

### Distance distribution within virus families

The number of virus pairs at each percentage of distance within each virus family was represented via a histogram. Pairs were represented in different colors according to their taxonomic relationships in NCBI’s taxonomy database: (1) the pair was colored in green if both genome sequences belong to the same species; (2) the pair was colored in yellow if the two genomes belong to different species but within the same genus; and (3) the pair was colored in pink if they belong to different genera.

### Taxonomic assignment for input virus sequences

VISTA is capable of performing taxonomic assignment of unknown viral genome sequences, which constitutes an important functionality of this tool. For each input sequence, VISTA first constructs a *k*-mer profile and extracts selected optimal features from it. Then a list of pairwise distances, from the lowest to the highest, is produced. The list consists of two types of distances: (1) distances between the input virus sequence and the rest of the input sequences, and (2) distances between the input virus and existing genomes within our reference database. The minimum distance is compared with demarcation thresholds (**Table 1**) by VISTA to determine the taxonomic relationship of the input virus with the closest genome sequence. If the minimum distance is below the species demarcation threshold within a virus family, the input virus is assigned to the same species as the closest sequence. If the minimum distance is above the species demarcation threshold but below the genus demarcation threshold, the input virus is assigned to a new species within the same genus. Otherwise, the input virus is assigned to a different genus. Specifically, within the class *Caudoviricetes*, if the minimum distance is above the family demarcation threshold, the input virus is assigned to a different virus family. The greater the distance from such taxonomic demarcation thresholds, the higher the confidence in the assignment of the input sequence.

**Table 1:** Optimal taxonomic demarcation thresholds for virus families analyzed in this study

Family	*k*-mer length	Genus demarcation threshold	FMI (genus)	Species demarcation threshold	FMI (species)	Distance
*Adenoviridae*	5	0.791	1.000	0.417	0.990	Standardized Euclidean
*Alloherpesviridae*	5	0.540	1.000	0.265	1.000	Normalized Google
*Alphaflexiviridae*	6	0.770	0.990	0.386	0.998	Euclidean
*Amalgaviridae*	5	0.676	1.000	0.171	1.000	Euclidean
*Arteriviridae*	5	0.653	1.000	0.353	1.000	Angled-based composition
*Astroviridae*	[3, 5]	0.796	1.000	0.376	0.912	Euclidean
*Baculoviridae*	6	0.791	1.000	0.163	0.939	Angled-based evolutionary
*Bornaviridae*	6	0.692	1.000	0.4	1.000	Standardized Euclidean
*Caulimoviridae*	6	0.921	0.996	0.437	0.959	Sokal-Sneath
*Circoviridae*	[3, 6]	0.517	0.993	0.256	0.987	Standardized Euclidean
*Coronaviridae*	6	0.709	0.998	0.518	0.995	Correlation
*Dicistroviridae*	5	0.747	1.000	0.335	1.000	Correlation
*Endornaviridae*	6	0.871	1.000	0.313	1.000	Normalized Google
*Filoviridae*	4	0.492	0.999	0.137	1.000	Correlation
*Flaviviridae*	6	0.725	0.996	0.406	0.985	Correlation
*Geminiviridae*	[3, 6]	0.865	0.988	0.391	0.746	Jaccard
*Hepadnaviridae*	5	0.653	1.000	0.445	0.985	Standardized Euclidean
*Hepeviridae*	6	0.452	1.000	0.281	0.977	Standardized Euclidean
*Herpesviridae*	[3, 6]	0.584	0.996	0.284	0.996	Squared Euclidean
*Iflaviridae*	6	–	–	0.204	0.987	Standardized Euclidean
*Inoviridae*	6	0.477	1.000	0.149	1.000	Correlation
*Iridoviridae*	[3, 5]	0.666	1.000	0.221	0.768	Euclidean
*Leviviridae*	5	0.767	1.000	0.423	1.000	Standardized Euclidean
*Microviridae*	5	0.748	1.000	0.292	0.888	Jaccard
*Mimiviridae*	5	0.782	1.000	0.151	1.000	Euclidean
*Papillomaviridae*	[3, 6]	0.703	0.974	0.391	0.964	Canberra
*Paramyxoviridae*	[3, 5]	0.674	0.999	0.351	0.987	Canberra
*Parvoviridae*	[3, 5]	0.608	0.950	0.215	0.949	Dice
*Phycodnaviridae*	6	0.762	1.000	0.025	0.707	Correlation
*Pneumoviridae*	5	0.770	1.000	0.487	1.000	Euclidean
*Polyomaviridae*	6	0.794	0.989	0.326	0.999	Euclidean
*Potyviridae*	5	0.704	0.999	0.261	0.962	Correlation
*Poxviridae*	5	0.508	1.000	0.071	0.548	Angled-based composition
*Rhabdoviridae*	6	0.452	0.997	0.120	0.994	Jensen-Shannon Divergence
*Tectiviridae*	5	0.639	1.000	0.146	1.000	Normalized Google
*Tombusviridae*	5	0.523	0.991	0.199	0.984	Jensen-Shannon Divergence
*Totiviridae*	6	0.822	0.938	0.259	0.976	Normalized Google
*Tymoviridae*	6	0.702	0.977	0.186	0.993	Dice

*Note*: FMI, Fowlkes-Mallows index.

## Results

### Description of VISTA

**Figure 1:**
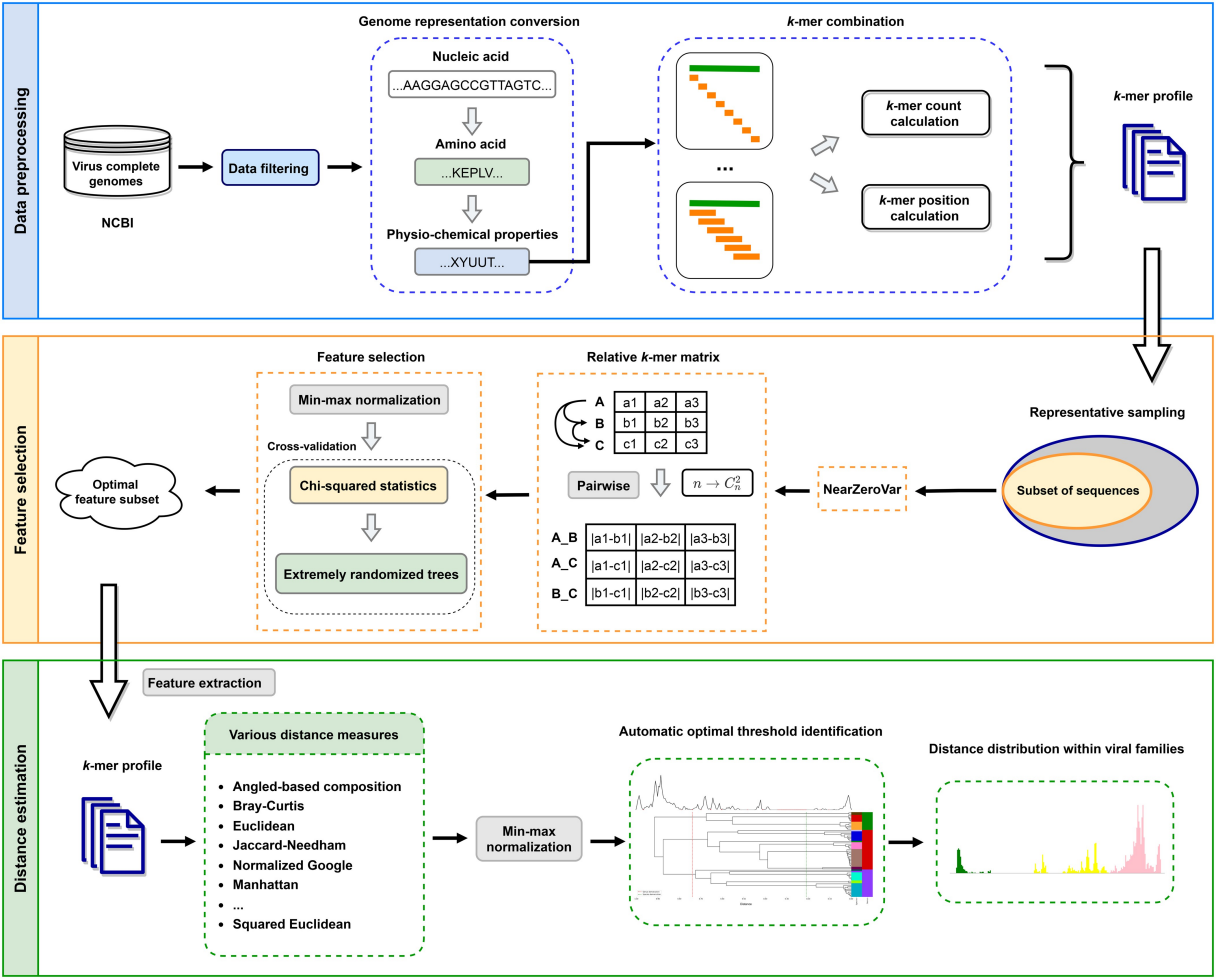
Schematic representation of VISTA workflow First, *k*-mer profiles were constructed by integrating frequency and position information of *k*-mers based on physio-chemical properties of amino acids to represent each complete viral genome sequence. Then, the *k*-mer features closely related to taxonomic relationships within the investigated virus family were selected for the following analysis. Lastly, a framework was designed for systematic and automatic identification of optimal distance distribution and taxonomic demarcation thresholds of the virus family. VISTA, Virus Sequence-based Taxonomy Assignment.

VISTA aims to reproduce the current taxonomy of virus families established by ICTV solely based upon sequences, and automatically assign new viral genome sequences into existing or novel taxa. Alignment-free methods are combined with machine learning techniques to generate pairwise distances between viral genomes. The VISTA workflow (**Figure 1**) consists of three major parts: (1) data preprocessing, (2) feature selection, and (3) distance estimation. Firstly, complete viral genome sequences were derived from the NCBI viral genomes collection [36], followed by a data filtering step to remove incomplete genome sequences and to decrease the redundancy in the dataset. A total of 21,263 complete virus sequences from 38 distinct virus families and the class *Caudoviricetes*, together with their NCBI taxonomy lineages, were retrieved for further analyses (Table S1). Next, we converted nucleotide sequences to physio-chemical property sequences, as these properties are more evolutionarily conserved and highly associated with protein function, structure, and classification [47–49]. Subsequently, *k*-mer profiles of the physio-chemical property sequences were constructed, integrating both frequency and positional information of *k*-mers to represent each viral genome sequence. Next, a two-step feature selection approach was employed to identify the optimal *k*-mer features that exhibit the strongest associations with different taxonomic relationships. This was done following representative sampling and relative *k*-mer conversion (see Method). Lastly, 19 different measures were applied for distance estimation. These measures vary in how they measure dissimilarity, and may have different assumptions and potential biases. Pairwise distances were created by the top performer from the 19 different distance estimation measures, and the pairwise distance distribution was generated for each virus group. We proposed a fully objective computational framework to automatically select optimal taxonomic demarcation thresholds. This also enables us to compare the performance of different distance measures quantitatively and choose the best one that introduces a minimal change in the current ICTV classification scheme for a specific virus group.

Below, we showcase how VISTA effectively tackles the challenges encountered by PASC in establishing demarcation criteria for specific viruses, and highlight its characteristics of high accuracy, high automation, and high speed in assigning viral genomes to established or new taxa.

### VISTA can produce better separation for taxonomic groups than PASC

**Figure 2:**
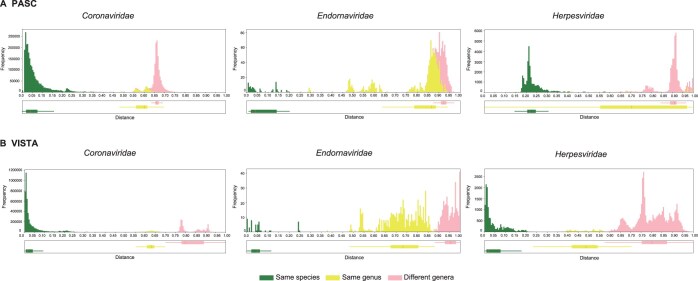
Comparison of distance distributions depicted by PASC and VISTA within the families *Coronaviridae*, *Endornaviridae*, and *Herpesviridae*Frequency distributions of pairwise distances from all complete genome sequences within three virus families (*Coronaviridae*, *Endornaviridae*, and *Herpesviridae*) generated by PASC (**A**) and VISTA (**B**). The color-coded bars represent pairwise taxonomic relationships: green (same species), yellow (different species but within the same genus), pink (different genera). The boxplot under each graph is to represent the distribution of distances for each category. PASC, Pairwise Sequence Comparison.

To facilitate comparison between VISTA and PASC, we transformed pairwise identities generated by PASC into pairwise identity distances (100% – identity %), which also range in value between 0 and 1. First, we applied VISTA to virus families with well-defined demarcation criteria by PASC (*Adenoviridae*, *Bornaviridae*, and *Filoviridae*) (Figure S2A). We observed clear separation of peaks for different taxonomic groups without any overlap (Figure S2B). This indicates that VISTA achieves comparable performance to PASC in cases where PASC performs well. We then focused on three virus families (*Coronaviridae*, *Endornaviridae*, and *Herpesviridae*) where establishing demarcation criteria using PASC is challenging. For the family *Coronaviridae*, there was a great deal of mixture of yellow and pink bars, with overlapping peaks ranging from 0.62 to 0.64 (**Figure 2**A). The yellow bars represent genome pairs that belong to different species but share the same genus, while the pink bars signify genome pairs that originate from different genera. This complex mixture suggests that PASC may encounter challenges in accurately determining the taxonomic assignments for virus pairs with identities situated in this particular region. Similarly, for the family *Endornaviridae*, most pink bars were mixed with yellow bars in the 0.88–0.94 range in PASC, lacking a clear threshold between these distinct colors. The family *Endornaviridae* only consists of two genera, *Alphaendornavirus* and *Betaendornavirus*, which are separated based on their different genome lengths, hosts, and unique domains [50]. However, PASC struggled to distinguish them using pairwise identities alone. For the family *Herpesviridae,* whose members are diverse and have large and complex genomes, most of the yellow bars were mixed with the other colors and did not form a cluster or peak, making it impossible to determine the genus demarcation. To evaluate whether VISTA could produce better separation for these groups, we applied it to the same virus families (Figure 2B). Remarkably, VISTA successfully minimized the mixing of bars of different colors, enabling the same taxonomic groups to cluster together and ensuring clear separation between different groups. This indicates that VISTA’s assignment of virus sequences aligns well with the current ICTV taxonomy, facilitating the establishment of species and genus demarcations for all three families. It is important to note that members of the three families exhibit significant variations in virion structure, host type, genome type, and genome size (Table S1), indicating the wide range of potential applicability of VISTA.

### VISTA can identify optimal taxonomic demarcation thresholds automatically

VISTA offers an automated approach to identify taxonomic demarcation thresholds objectively. To exemplify its practical utility, we present an instance of taxonomic demarcation threshold identification for the family *Endornaviridae*.

**Figure 3:**
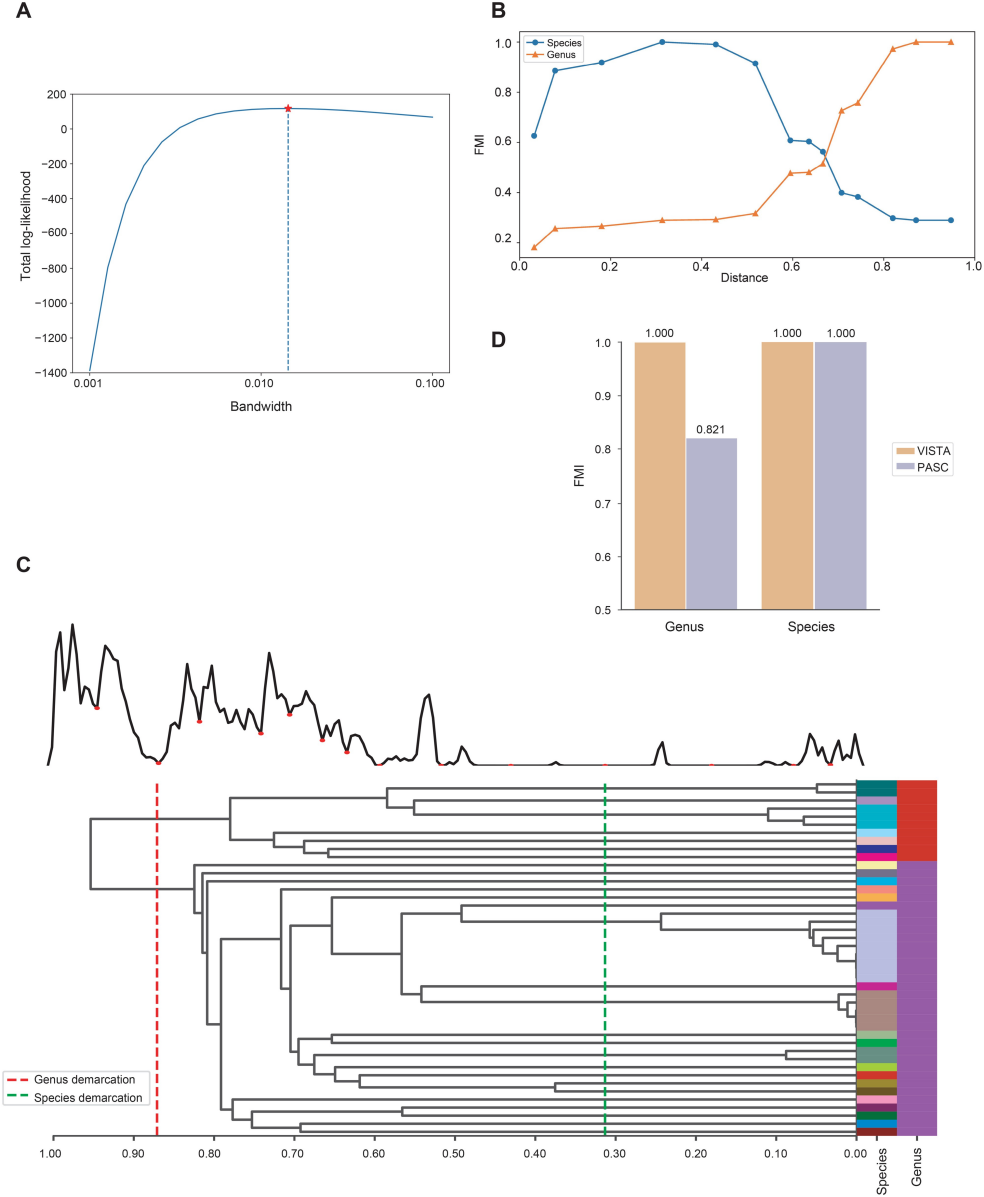
Identification of taxonomic demarcation thresholds for the family *Endornaviridae***A**. Bandwidth selection for kernel density estimation. The bandwidth was optimized via a grid search cross-validation approach. **B**. Evaluation of optimal distance thresholds for the family *Endornaviridae*. The X-axis denotes all detected thresholds (local minima) in the distance distribution from dist = 0 to dist = 1. The Y-axis denotes the FMI scores when trying to recapitulate ICTV genera and species. From these data, distances of 0.313 and 0.871 yielded the highest FMI scores for the species-rank and genus-rank viral clusters, respectively. **C**. Threshold candidates are labeled with red dots on the density distribution of pairwise distances. Besides, a full-link dendrogram is displayed, where each row represents a viral genome sequence and the column with a unique color represents the genome’s ICTV species or genus. The thresholds which achieve the highest FMI scores are recognized as taxonomic demarcation thresholds for the virus family. **D**. Comparison of overall clustering performance of VISTA and PASC for the family *Endornaviridae*. FMI, Fowlkes-Mallows index; ICTV, International Committee on Taxonomy of Viruses.

First, we employed Gaussian kernel density estimation to fit pairwise distances and optimized the bandwidth using cross-validation. Unlike DEmARC which fits data using a normal mixture model, the kernel density estimation we used does not require any parametric assumptions on the true density and can be applied to a broader range of probability distributions [51,52]. The optimal bandwidth of 0.01438 was determined by achieving the highest total log-likelihood score (**Figure 3**A). After the fitting step, valleys of the distribution were identified, as they are expected to generate a very low degree of conflict or uncertainty when assigning newly sequenced viruses [18,53,54]. Meanwhile, hierarchical clustering was performed based on all intergenomic distance values for the family *Endornaviridae*. We applied different distance thresholds, obtained from the valley list, to the hierarchical tree to divide viral genome sequences into clusters at various taxonomic ranks. The FMI was used to evaluate the suitability of the thresholds to define genus- and species-rank clusters (Figure 3B). The FMI score curves for both species- and genus-rank clusters initially increased and then either decreased or stabilized as the distance threshold increased. The highest FMI score for species-rank clusters was achieved at a distance of 0.313, while a distance of 0.871 attained the highest score for genus-rank clusters. These distance values successfully delineate all species and genera within the family *Endornaviridae* (Figure 3C). Therefore, we considered distances of 0.313 and 0.871 as the optimal species and genus demarcation thresholds, respectively.

By comparing the highest FMI scores, we can not only select optimal thresholds but also quantitatively evaluate the clustering performance of different methods. We conducted a comparison between VISTA and PASC based on their highest FMI scores (Figure 3D). VISTA achieved perfect FMI scores of 100.0% at both the genus and species ranks, while PASC attained 82.1% and 100.0%, respectively. Although PASC performed well at the species rank, it obtained a significantly lower FMI score at the genus rank compared to VISTA. This result is consistent with the comparison of distance distributions within the *Endornaviridae* family (Figure 2), demonstrating that VISTA’s inferred genus-rank clusters align more closely with the current ICTV taxonomy than those derived from PASC. In this study, we have successfully identified optimal taxonomic demarcation thresholds for 38 virus families using VISTA (Table 1).

### Application of VISTA to tailed phages in the class *Caudoviricetes*

To adapt to the latest ICTV phage classification system, we abandoned previous models based on the three virus families *Myoviridae*, *Podoviridae*, and *Siphoviridae* and instead directly utilized VISTA for *Caudoviricetes*. We obtained all complete viral genome RefSeq sequences within *Caudoviricetes* from the NCBI Viral Genomes Resource on January 1, 2023. Additionally, all exemplar viruses from the Master Species List (MSL37) and the Virus Metadata Resource (VMR37) [55] were retrieved, and the final dataset included 3655 genome sequences. In *Caudoviricetes*, many sequences have incomplete taxonomic information, with some being assigned to “unclassified” at the family rank, some at the genus rank, and even some directly listed under *Caudoviricetes* without any further taxonomic assignments. Unclassified viruses are typically dissimilar to currently known viruses, but it becomes difficult to determine their taxonomic relationships when two sequences are both unclassified at the family or genus rank. Therefore, we labeled these sequence pairs as “uncertain” and excluded them from the calculation of FMI scores. Next, to avoid excessive memory consumption, we randomly selected 600 sequences with definite family and genus-labels from the reference dataset for feature selection.

**Figure 4:**
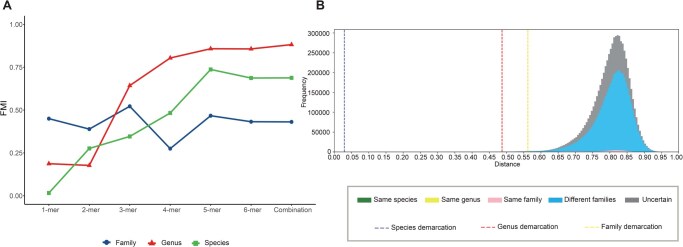
Performance of VISTA on the class *Caudoviricetes***A**. The line chart shows the impact of *k*-mer length variation and the combination of *k*-mers on clustering performance. **B**. The frequency distribution of pairwise distances generated by VISTA within the class *Caudoviricetes*. The color-coded bars represent pairwise taxonomic relationships: green (same species), yellow (different species but within the same genus), pink (different genera but within the same family), blue (different families), and gray (uncertain taxonomic relationships). The species, genus, and family demarcation thresholds (determined by VISTA) are indicated by blue, red, and golden dashed lines, respectively.

Considering that *Caudoviricetes* contains sequence pairs from different virus families, which may result in a different *k*-mer distribution pattern compared to that within a virus family, a range of *k*-mer lengths and combinations were retested (**Figure 4**A). The results showed that the change in clustering performance varied across different taxonomic ranks. At the species rank, the clustering performance rapidly improved with increasing k and reached the highest at k=5, then gradually decreased and tended to stabilize. At the genus rank, the clustering performance also improved with increasing k and began to stabilize at k=5, with a slight improvement thereafter. At the family rank, the performance exhibited more fluctuation. It was at a relatively high level when k=1, but dropped to the lowest point at k=4, then rose again at k=5, and then slightly decreased and tended to stabilize. Based on these results, we determined that the optimal *k* value for constructing the pairwise distance distribution within *Caudoviricetes* is 5. By applying the optimal *k* value of 5, we observed that the green bars clustered together, while most of the yellow bars were well separated from the green and pink bars (Figure 4B). The blue bars (representing different families) and the gray bars (representing uncertain relationships) formed a distinct peak within the range of 0.55 to 0.95. However, between 0.75 and 0.85, there were many pink bars mixed with the blue and gray peaks, indicating the discrepancy between the VISTA assignments and the current ICTV classification. Finally, we determined distances of 0.029, 0.487, and 0.562 as the optimal species, genus, and family demarcation thresholds, respectively.

### Taxonomic assignment for unknown viral genome sequences

To assess the reliability of our method in providing taxonomic assignments for unknown virus sequences, we applied VISTA to complete viral genomes that were not presented in our reference database. A total of 47 RefSeq viral genome sequences within *Caudoviricetes* were obtained from the NCBI Viral Genomes Resource between January 2 and March 1, 2023. These sequences had been given definite labels at each taxonomic rank, which allowed us to compare the assignment results obtained by VISTA with the current taxonomic assignments and determine the accuracy of our method. We considered two possible outcomes for the assignment results. (1) If a sequence was assigned to a novel family/genus/species and its corresponding taxonomic name did not exist in our reference database, the assignment was considered correct. Otherwise, it was considered an incorrect assignment. (2) If a sequence was assigned to a known family/genus/species and its corresponding taxonomic name matched the closest match found by VISTA, the assignment was considered correct. Otherwise, it was considered incorrect. The accuracy was calculated as the proportion of correct assignments.

Our results (Table S2) showed that out of the 47 input virus sequences, 42 had consistent assignments with the current RefSeq taxonomy annotations, yielding an overall VISTA assignment accuracy of 89.36% (42/47). Specifically, for taxonomic assignments at the family rank, VISTA achieved an accuracy of 93.62% (44/47). Among the misassigned sequences, we found that the minimum matching distances of NC_070859 and NC_070910 were located around the family/genus thresholds, which represent relatively ambiguous regions. Additionally, we speculate that some viruses might be assigned to an incorrect lineage by sequence submitters, leading to inconsistent assignment results.

To further validate the capability of our method in high-throughput discovery and taxonomic identification of novel viruses derived from metagenomic data, we applied VISTA to 679 unannotated and unclassified complete metagenome-assembled prokaryotic virus genomes obtained from a freshwater spring bloom (BioProject ID: PRJEB52406) [56]. Through the analysis of generated distances between inputs and our reference database, we observed that all sequences should be assigned to novel viruses. The majority of these sequences belonged to new families, but most of them originated from the same family. VISTA’s results suggest that up to 46 new families may be potentially defined based on these assignments (Table S3).

### Runtime comparison between VISTA and PASC

The runtime for pairwise sequence comparison methods is influenced by the size of input data and the number of sequences that need to be compared. To assess the computational efficiency of VISTA and PASC, we selected three different virus families with varying numbers of sequences: *Dicistroviridae* (N=114), *Circoviridae* (N=1680), and *Coronaviridae* (N=14,676). We measured the runtimes for both methods using different sizes of input data. We randomly sampled n (n=1, 10, 50, 100) sequences from each virus family as input and repeated this process 3 times to obtain the average runtime. Our results showed that VISTA was considerably faster than PASC in all three families, with significant speed advantages. In the case of the *Coronaviridae* family, VISTA was up to 110 times faster than PASC (**Table 2**). Furthermore, PASC failed to run when the number of input sequences exceeded 50, and this limitation reduced to only one sequence when applied to the *Coronaviridae* family. In contrast, VISTA did not have a maximum data size limitation of 50, and it efficiently performed taxonomic assignments even with over 100 sequences within a reasonable timeframe.

**Table 2:** Runtime comparison between PASC and VISTA

	*Dicistroviridae*				*Circoviridae*				*Coronaviridae*			
	*n* = 1	*n* = 10	*n* = 50	*n* = 100	*n* = 1	*n* = 10	*n* = 50	*n* = 100	*n* = 1	*n* = 10	*n* = 50	*n* = 100
PASC	23.00 s	68.00 s	483.33 s	–	49.33 s	378.00 s	1816.00 s	–	1725.33 s	–	–	–
VISTA	6.67 s	20.00 s	111.00 s	249.67 s	9.00 s	37.33 s	217.33 s	483.67 s	15.00 s	78.33 s	421.67 s	984.33 s

*Note*: *n* represent the number of randomly sampled sequences from each virus family.

### Web interface and standalone application

**Figure 5:**
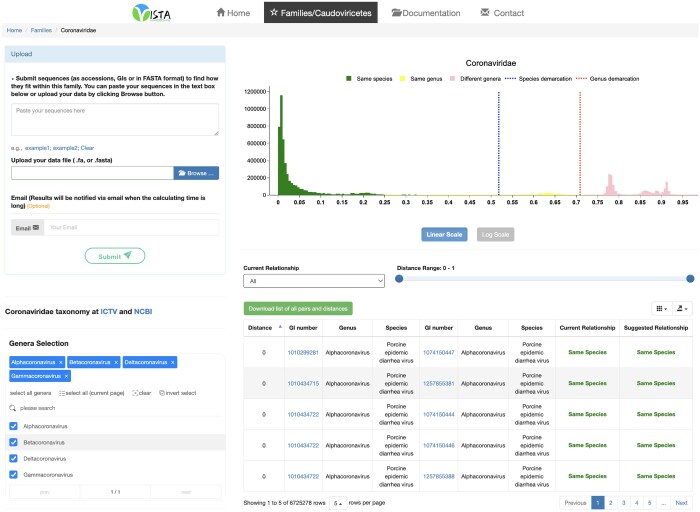
Screenshot of VISTA displaying the result of pairwise distance distribution within a virus familyThe pairwise distance distribution together with identified optimal taxonomic demarcation thresholds is displayed. Users can either submit DNA sequences in FASTA format or paste the sequence or Accession/GI number directly into the text box for taxonomic assignments.

To facilitate the implementation of our method, we developed a user-friendly web-based interface available at https://ngdc.cncb.ac.cn/vista. The interface offers an interactive platform for users to explore the relationships between different taxa based on the pre-computed pairwise distances and perform taxonomic assignments for genome sequences of interest (**Figure 5**). Users can submit their genome sequences by pasting text or uploading a file in FASTA format without adjusting any parameters, and the tool will directly provide the results of taxonomic assignments for each sequence. For those who prefer to use VISTA locally, the entire pipeline was wrapped within a Docker container, with software versions pinned for reproducible execution (https://hub.docker.com/r/taozhangbig/vista). By downloading the Docker image and running VISTA via command lines on their local machines, users can experience the convenience and reproducibility of VISTA without relying on the web-based interface. Moreover, VISTA can run in local multithreading mode, further enhancing its performance and efficiency for large-scale analyses.

## Discussion

In this work, we present VISTA, a dedicated tool for virus taxonomic assignment based on a novel pairwise sequence comparison system. Unlike PASC, which relies on the BLAST-based alignment for calculating identities between genome sequences, VISTA utilizes an alignment-free method complemented by machine learning techniques to enhance its performance. This has broadened the applicability of VISTA, enabling its effective utilization across a wider range of viruses and establishing it as a more versatile and adaptable assignment tool compared to PASC.

A notable feature of VISTA is its ability to identify viruses with incorrect taxonomic assignments in the existing database. In instances where submitters have assigned their sequences to incorrect virus names, such misassignments can lead to a color mixture in the resulting peaks. For example, the genome sequence NC_018703 (Culex originated Tymoviridae-like virus) was assigned to the phylogenetically closest genus *Maculavirus*, even though its molecular characteristics were distinct from those of tymoviruses, marafiviruses, maculaviruses, or macula-like viruses [57]. However, by using VISTA, we discovered that the pairwise distances between this virus and existing maculaviruses were all above the established genus demarcation threshold, which resulted in some yellow bars mixed with the pink dominant peak in the 0.78–0.89 range (Figure S3). The output from VISTA provided compelling evidence that NC_018703 should be reassigned to a novel genus within the family *Tymoviridae*.

VISTA presents several distinct advantages over PASC. Firstly, it demonstrates improved separation capabilities for distinguishing between different ICTV-ratified genera. For example, despite the distinct internal ribosomal entry site topology and phylogenetic divergence between the genera *Triatovirus* and *Cripavirus* in the family *Dicistroviridae* [58], PASC struggled to differentiate them due to overlapping sequence identity distances (Figure S4A). Similarly, within the family *Tombusviridae*, PASC output displayed considerable overlaps between the intergeneric and intrageneric distances for *Alphanecrovirus* and *Gammacarmovirus* (Figure S4A), even though these two genera were assigned by a phylogenetic analysis based on the RNA-directed RNA polymerase (RdRP) protein and the coat protein (CP) [59,60]. In contrast, VISTA effectively captured the divergences between these genera, generating pairwise distance distributions with distinct valleys that clearly separated the different colored bars (Figure S4B). Secondly, VISTA introduces a novel strategy to assign highly divergent viruses. While whole-genome alignment methods based on nucleotide or amino acid sequences can effectively capture divergence between very similar viruses, they often fall short when applied to highly divergent viruses or large DNA viruses displaying extensive genome rearrangements (*e.g.*, *Herpesviridae*) [61]. In such cases, taxonomic assignment based on short motifs may be a more suitable choice. Consequently, VISTA employs physio-chemical property sequences, which are more evolutionary conserved than nucleotide or amino acid sequences, and combines different sizes of *k*-mers to capture distribution patterns of different lengths of short sequence motifs analogically. Our analysis of different virus families reveals that the *k*-mer combination can further improve clustering performance for some divergent viruses (Figure S1B). In addition, the positions of *k*-mers were also incorporated, which could be informative to detect genomic rearrangement events, such as inversion, transposition, translocation, and recombination. Importantly, we demonstrate that these approaches, in combination with a series of feature selection methods, can achieve superior performance and higher scalability compared to PASC. Particularly, we applied VISTA to analyze tailed phages with highly divergent genome sequences and identified optimal thresholds for species, genus, and family demarcation within the class *Caudoviricetes*. The output rank of VISTA has been extended to the family rank, which is not achievable with PASC. Thirdly, VISTA introduces an objective framework for automatically determining optimal taxonomic demarcation thresholds without manual intervention. This approach streamlines the taxonomic assignment process and eliminates subjective biases that may arise from manual intervention. Lastly, VISTA is a readily accessible bioinformatic tool, available through both a user-friendly web interface and a standalone program. Its exceptional speed, ease of use, and high rank of automation make it well-suited for high-throughput virus taxonomic assignment.

However, there are limitations in VISTA. One limitation is the potential bias toward virus taxa with a large number of sequences. Overrepresented viruses tend to exert a dominant influence on the selection of the final feature subset, while underrepresented viruses contribute relatively little, especially for species or genera with only a single sequence. This would make it difficult for VISTA to distinguish them using the selected features if their genomes lack obvious distinguishing characteristics. For instance, within the family *Alphaflexiviridae*, the genus *Lolavirus* currently consists of just one complete genome sequence (Lolium latent virus) [62]. Consequently, our approach fails to effectively differentiate the genus *Lolavirus* from the dominant genus *Potexvirus*. In contrast, this is not a concern for PASC, as its alignment-based approach is less sensitive to the number of samples. It is worth noting, however, that as the number of members in such taxonomic groups increases, this limitation may be mitigated in the future. Additionally, the handling of segmented viruses represents another challenge. Similar to the PASC methodology, VISTA could potentially address this by treating each genome segment as a separate “virus” sequence and then conducting individual analyses. However, this approach is not yet integrated into VISTA and could be considered for inclusion in the future. Furthermore, although we have established a taxonomic assignment model for *Caudoviricetes*, there are still discrepancies between this model and the current ICTV classification system, especially at the rank of different families and different genera within the same family. The results indicate a need for further refinement of our approach to enhance its adaptability. Simultaneously, considering the fluid nature of phage taxonomy, the observed inconsistencies may warrant a comprehensive reevaluation of the current taxonomic classifications by experts. Finally, we need to regularly update our reference database and further retrain our model based on the latest ICTV changes to keep up with the evolving field of viral taxonomy.

In the future, we will be dedicated to further exploring the potential of VISTA, and maximize its application for as many viruses as possible. If widely accepted, VISTA could represent a significant step toward the realization of a universal tool for genome-based viral taxonomy.

## Code availability

The package of VISTA has been submitted to BioCode at the National Genomics Data Center (NGDC), China National Center for Bioinformation (CNCB), which is publicly available at https://ngdc.cncb.ac.cn/biocode/tool/7578.

## Supplementary Material

qzae082_Supplementary_Data

## Data Availability

The data used to support the findings of this study are available from VISTA’s web site at the NGDC (https://ngdc.cncb.ac.cn/vista).

## CRediT author statement

**Tao Zhang:** Investigation, Methodology, Software, Writing – original draft, Writing – review & editing. **Yiyun Liu:** Data curation, Formal analysis, Investigation, Writing – review & editing. **Xutong Guo:** Formal analysis, Validation. **Xinran Zhang:** Formal analysis, Validation. **Xinchang Zheng:** Software. **Mochen Zhang:** Validation. **Yiming Bao:** Conceptualization, Supervision, Project administration. All authors have read and approved the final manuscript.
