# Supplementary material for: VISTA: A Tool for Fast Taxonomic Assignment of Viral Genome Sequences
Source: Genomics Proteomics Bioinformatics. 2024 Nov 14;23(1):qzae082. doi: 10.1093/gpbjnl/qzae082 (PMC12212643; doi:10.1093/gpbjnl/qzae082)
Supplement: qzae082_Supplementary_Data [file qzae082_supplementary_data.zip › supplementary material captions.docx]

# Supplementary material

**Figure S1 The impact of *k*-mer length and *k*-mer combination on the performance of VISTA**

**A.** The boxplots show how clustering performance improves with increasing the *k*-mer length for 10 representative virus families (*Poxviridae*, *Mimiviridae*, *Papillomaviridae*, *Totiviridae*, *Filoviridae*, *Alphaflexiviridae*, *Phycodnaviridae*, *Coronaviridae*, *Hepadnaviridae*, and *Inoviridae*). **B.** Comparisons of clustering performance between using the *k*-mer combination and using one fixed-length *k*-mer.

**Figure S2 Comparison of distance distributions depicted by PASC and VISTA within the families *Adenoviridae*, *Bornaviridae*, and *Filoviridae*.**

Frequency distributions of pairwise distances from all complete genome sequences within three virus families (*Adenoviridae*, *Bornaviridae*, *Filoviridae*) generated by PASC (**A**) and VISTA (**B**). The bars colored in green represent that both genomes belong to the same species; in yellow represent that the pair of genomes belong to different species but the same genus; and in pink represent that they belong to different genera.

**Figure S3 Screenshot of the assignment result for the sequence NC_018703 generated by the VISTA web server**

**Figure S4 Comparison of distance distributions depicted by PASC and VISTA between members of different genera**

Analysis of two dicistrovirus genera (left) and two tombusvirus genera (right) by PASC (**A**) and VISTA (**B**).

**Table S1 Viruses analyzed in this study and their associated information**

**Table S2 Taxonomic assignments for unknown viral genomes in the class *Caudoviricetes***

**Table S3 Taxonomic assignments for 679 unclassified complete metagenomic-assembled prokaryotic virus genomes**
