# Supplementary figures and images for: VISTA: A Tool for Fast Taxonomic Assignment of Viral Genome Sequences
Source: Genomics Proteomics Bioinformatics. 2024 Nov 14;23(1):qzae082. doi: 10.1093/gpbjnl/qzae082 (PMC12212643; doi:10.1093/gpbjnl/qzae082)

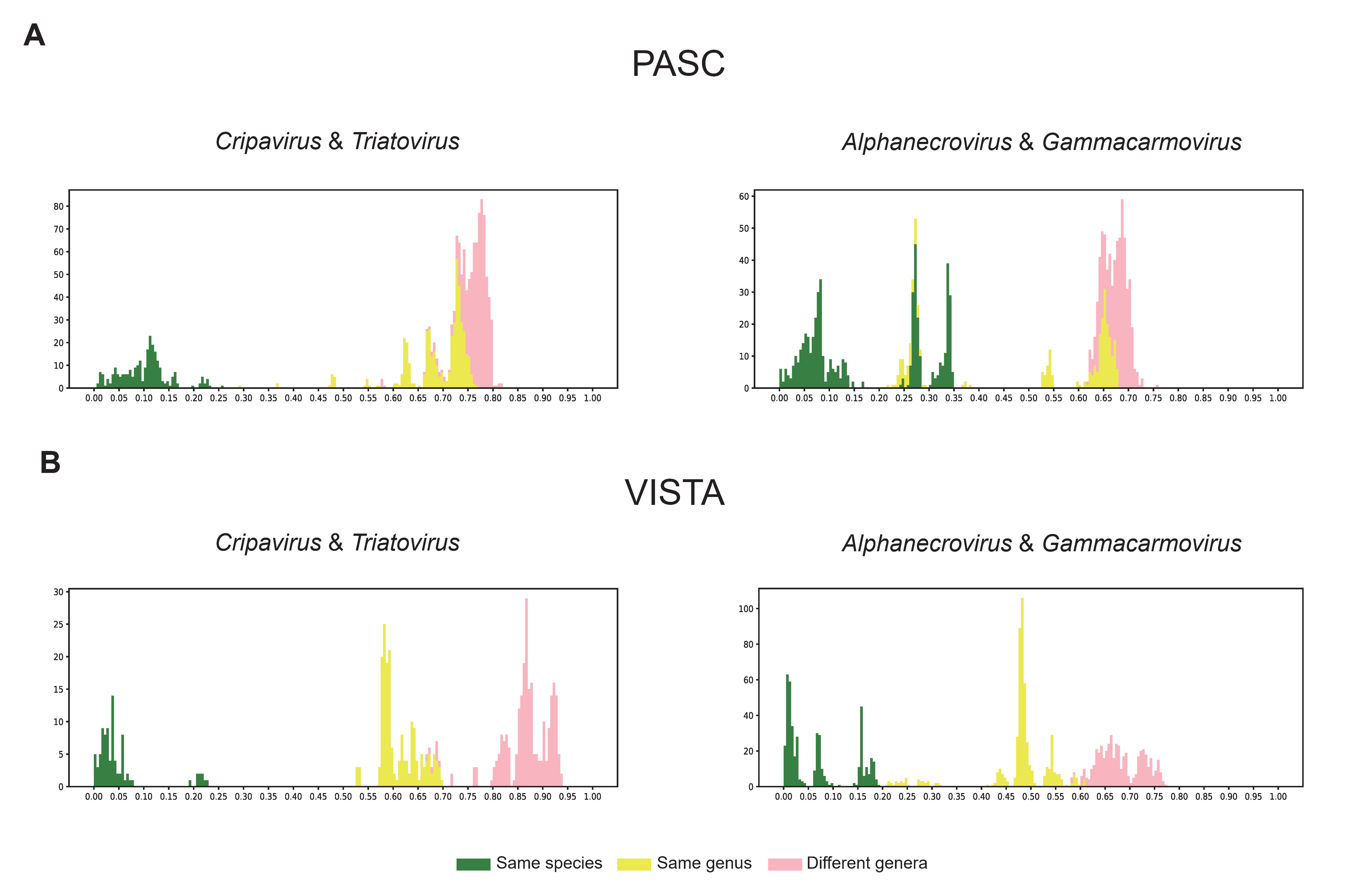

Supplement: qzae082_Supplementary_Data [file qzae082_supplementary_data.zip › Supplementary Figure 4.jpg]

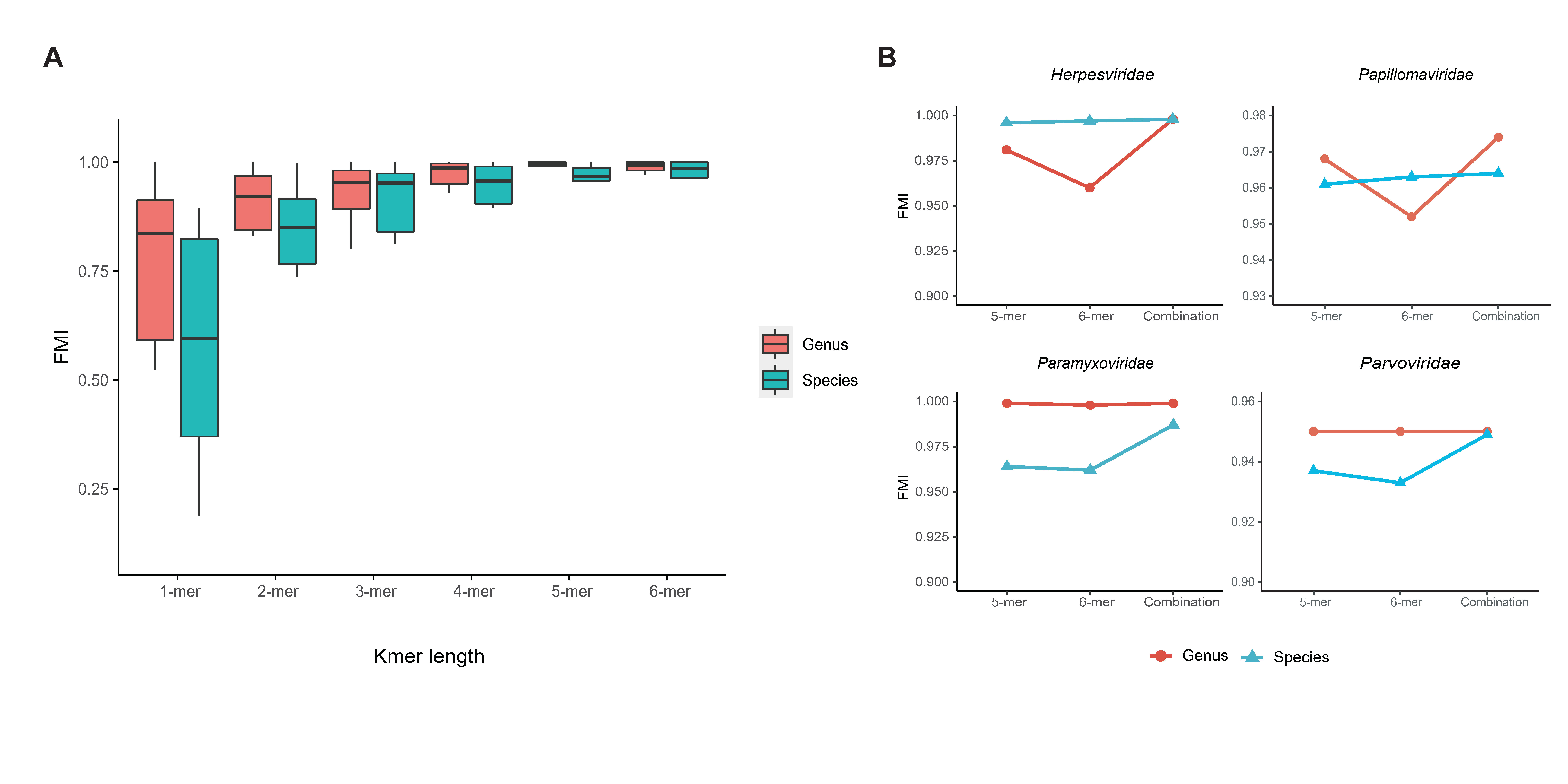

Supplement: qzae082_Supplementary_Data [file qzae082_supplementary_data.zip › Supplementary Figure 1.jpg]

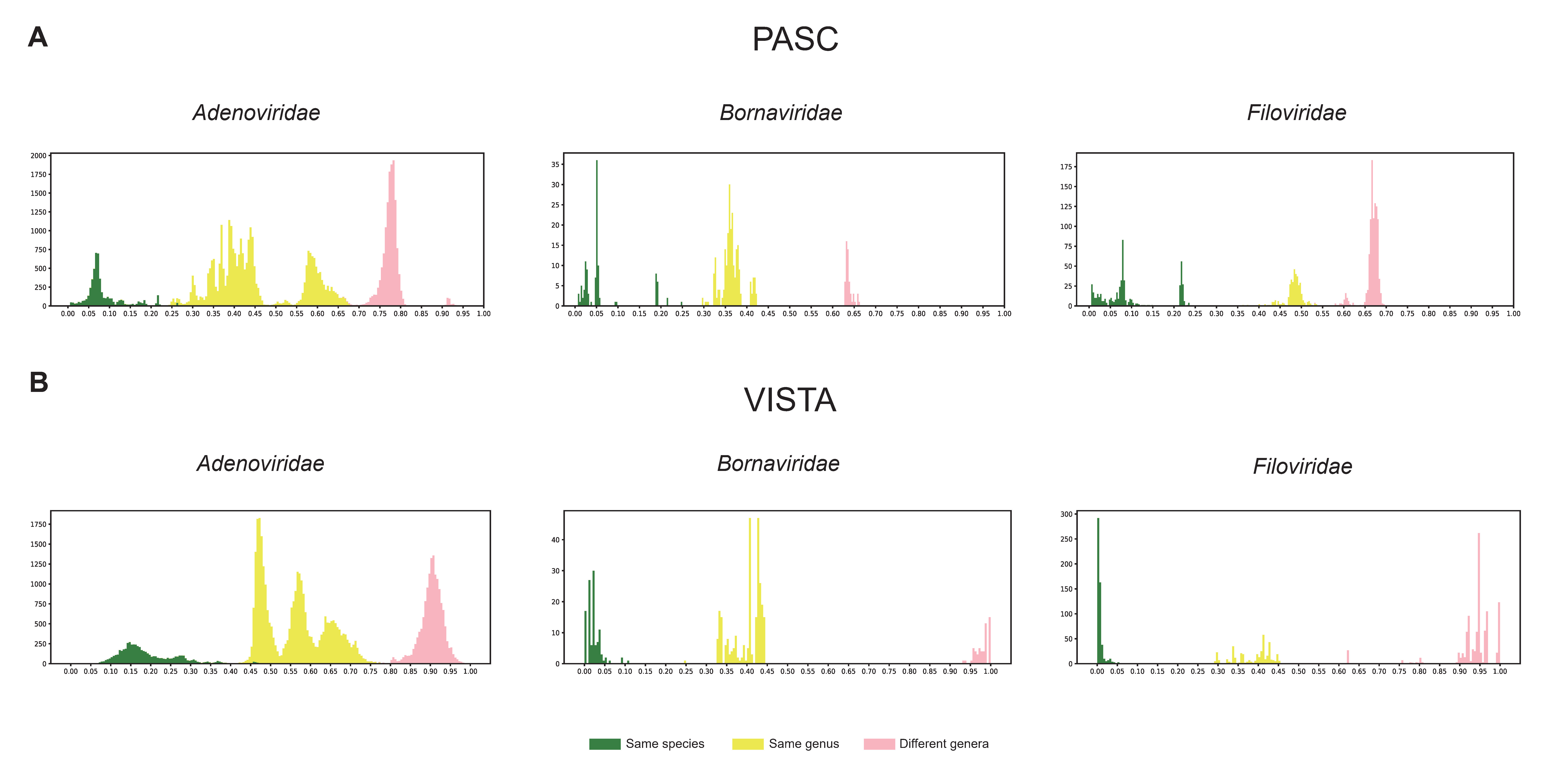

Supplement: qzae082_Supplementary_Data [file qzae082_supplementary_data.zip › Supplementary Figure 2.jpg]

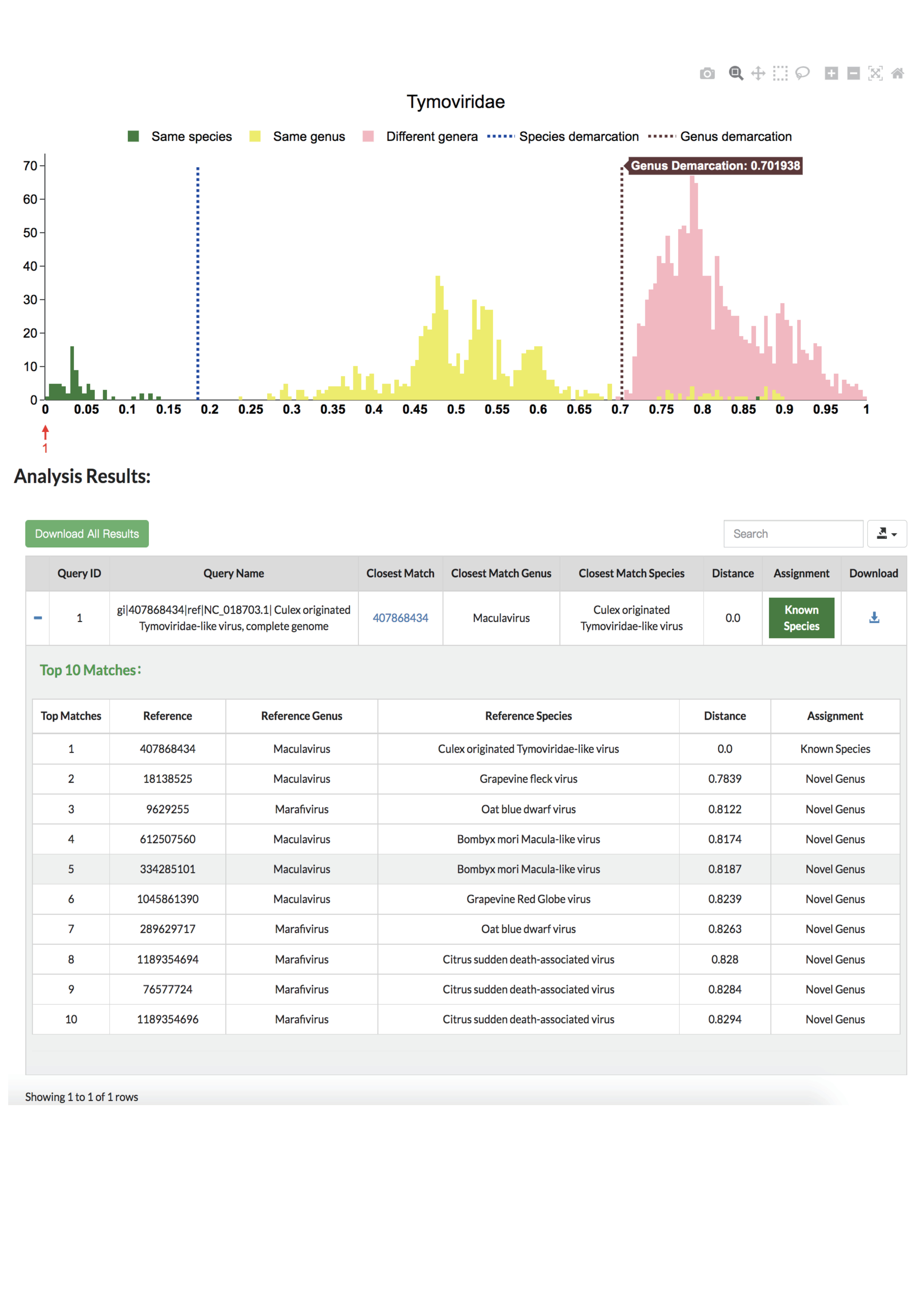

Supplement: qzae082_Supplementary_Data [file qzae082_supplementary_data.zip › Supplementary Figure 3.jpg]
